# Prognostic Value of Early Rehospitalization in Heart Failure Patients

**DOI:** 10.3390/jcm14217720

**Published:** 2025-10-30

**Authors:** Sergey Panov, Kristina Charaya, Sofya Sovetova, Dmitry Shchekochikhin, Shevket Ibraimov, Alexandra Bogdanova, Elena Mashkova, Sofiia Lomakina, Nina Novikova, Abram Syrkin, Denis Andreev

**Affiliations:** 1 Department of Cardiology, Functional and Ultrasound Diagnostics, First State Moscow University Named After I.M. Sechenov, Trubetskaya 8/2, 119991 Moscow, Russia; charaya9716@gmail.com (K.C.); sovetovasofja@gmail.com (S.S.); agishm@list.ru (D.S.); sheva7864@gmail.com (S.I.); doc.aabogdanova@gmail.com (A.B.); sonyalom@yandex.ru (S.L.); novikova_n_a@staff.sechenov.ru (N.N.); syrkin_a_l@staff.sechenov.ru (A.S.); dennan@mail.ru (D.A.); 2 City Clinical Hospital Named After S. S. Yudin, Kolomensky Passage 4, 115446 Moscow, Russia; 3 City Clinical Hospital No. 1 Named After N.I. Pirogov, Leninsky Prospekt 8, 119049 Moscow, Russia; 4 City Polyclinic No. 5, Bolshaya Bronnaya St., Building 3, 123104 Moscow, Russia; dr.kardio@bk.ru

**Keywords:** acute heart failure, cause of readmission, early readmission, diuretics

## Abstract

**Background:** Acute heart failure (AHF) is a common cause of hospital admission with high morbidity and mortality. Up to one-third of AHF patients require rehospitalization during the first three months after discharge due to the nature of disease and the patient’s characteristics. In this regard, the first 3 months after an episode of decompensation of heart failure are called the “vulnerable” period. However, there is a gap in knowledge about the significance of this rehospitalization on heart failure course. The aim of the study is to evaluate impact on mortality of AHF rehospitalization during 3 months after hospital discharge on a retrospective registry with 3 year follow-up. **Methods:** Patients after AHF hospitalization episode between 1 December 2020 and 30 November 2023 were monitored via electronical medical records for 3 year follow-up. All patients who survived after index hospitalization were included. The primary endpoint was all-cause mortality. COX-multiple regression was used to evaluate the impact of rehospitalization during 90 days after index discharge on outcomes. *p* values less than 0.05 were considered to be significant. **Results:** A cohort of 204 patients, 56.6% males, with an average age of 72 ± 13 years, were included in the study with medium follow-up of 22 ± 12 months. Within 3 months after discharge, 55 (27%) patients were rehospitalized for AHF, and 11 (5%) patients died. Patients who experienced a recurrent episode of AHF were characterized by a history of previous hospitalizations for AHF before inclusion (39 (71%) vs. 72 (48%); *p* = 0.005), the use of intravenous inotropic drugs (5 (9%) vs. 2 (1%); *p* = 0.007), higher initial doses of furosemide during index hospitalization (98 ± 46 vs. 82 ± 37; *p* = 0.01), and higher doses of furosemide at discharge (54 ± 41; 41 ± 33; *p* = 0.02). Left ventricular ejection fraction (LVEF), prevalence of atrial fibrillation (AF), diabetes mellitus (DM), and chronic kidney disease (CKD) did not differ between the groups. Over 3 years follow-up, 68 (33.2%) patients died, and cardiovascular mortality was 15.6% (32 patients). In multivariate COX-regression age (HR 1.04 [1.008–1.07]), heart rate (HR) on admission (HR 1.02 [1.004–1.03]), and hospitalization within the first 3 months after discharge were independent predictors of death (HR 2.21 [1.32–3.83]). **Conclusions:** Readmission for AHF within the first 3 months after discharge is an independent risk factor for all-cause cardiovascular mortality during 3 years follow-up.

## 1. Introduction

Heart failure (HF) is a disease accompanied by typical symptoms (shortness of breath, weakness, edema) and signs (swelling of the jugular veins, gallop rhythm, moist rales in the lungs), which proceeds in waves, where periods of stable progression alternate with periods of deterioration in the condition—acute heart failure (AHF) [1].

Despite significant progress in the treatment of CHF, each episode of AHF affects the prognosis of the disease, increasing the risk of rehospitalization and mortality [2]. This risk remains particularly high during the first three months after discharge: it is known that the prevalence of rehospitalizations during this period is about 30%, and mortality can approach 10%. Due to the high risk of adverse outcome, the first 3 months after an episode of AHF are defined as a “vulnerable period” for these patients [3].

Each episode of AHF may be not only a deterioration in the physical condition and cardiac function of patients with HF but also a decrease in adherence to treatment, which creates the preconditions for further deterioration of the prognosis [4].

Despite the recognized unfavorable role of AHF episodes in the course of HF, the nature of the relationship between the episode of rehospitalization during vulnerable period and long-term prognosis has not been evaluated essentially. The aim of this study is to assess the prognostic value of rehospitalization due to AHF within 3 months after discharge during long-term observation.

## 2. Materials and Methods

A retrospective single-center study was conducted with an analysis of electronic medical records of hospitalized AHF patients between 1 December 2020 and 30 November 2023. Entire cohorts of AHF patients were presented in previous publication [5,6]. For further analysis, these patients were observed via electronical medical records in a prospective manner up to 3 years follow-up.

The study was approved by the local ethics committee of Sechenov University extract No. 29-24 dated 5 December 2024.

Patients who required rehospitalization due to AHF during the first three months after discharge formed the main group. Others were used as a control. All-case mortality was a primary end point. All quantitative variables were tested for normal distribution using the Kolmogorov–Smirnov test. Variables with normal distribution were described by the mean and standard deviation. In case the distribution differed from normal, it was described by the median and interquartile range between the 25th and 75th percentile and compared using nonparametric tests. To compare groups by quantitative variables, Student’s *t*-test (under normal distribution) or Mann–Whitney test (under non-normal distribution) was used. Categorical variables were presented as absolute and relative values, and the chi-square test or Fisher’s test were used to compare them. COX analysis was used to assess the influence of risk factors on prognosis. SPSS 22.0 for Windows (SPSS Inc., Chicago, IL, USA) was used for statistical analysis. Differences were considered statistically significant at *p* < 0.05.

## 3. Results

**Figure 1:**
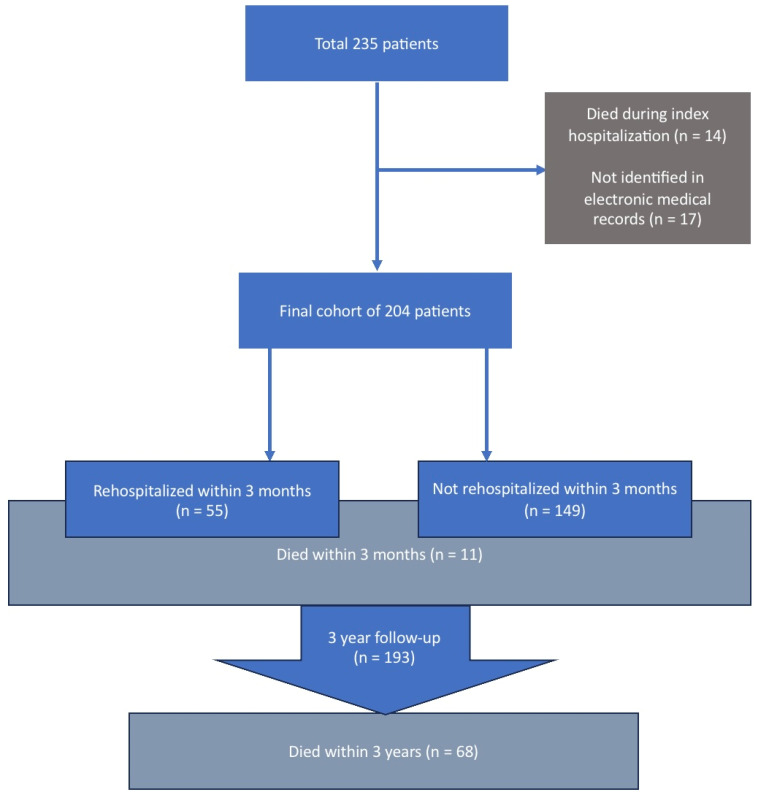
Study design.

The initial database included 235 patients. After excluding patients who died during the index hospitalization (14 patients) and those not identified in the unified medical system (17 patients), the final sample included 204 patients. The flow-chart is presented in Figure 1.

The mean age was 72 ± 13 years, and 116 (56.6%) patients were men. A total of 75 (36.8%) patients required hospitalization in the intensive care unit (ICU).

The main cardiac comorbidities were CAD; 108 (52.7%) patients had a history of myocardial infarction (MI), and atrial fibrillation (AF) was observed in 148 (72.2%) patients. Moreover, 134 (65.4%) patients had AF at the time of admission. Main non-cardiac comorbidities were diabetes mellitus (DM) in 59 (28.8%) patients, chronic obstructive pulmonary disease (COPD) in 44 (21.5%) patients, and chronic kidney disease (CKD) stages 3b and 4 in 75 (36.8%) patients.

Average left ventricular ejection fraction (LVEF) was 43 ± 16%; in 121 (59%) patients, LVEF was less than 40%.

According to discharge summaries, 179 (87.3%) patients received angiotensin-converting enzyme inhibitors (ACEIs) or ARB, beta-blockers (BB) were received by 155 (75.6%) patients, mineralocorticoid antagonists (MCA) spironolactone or eplerenone were received by 162 (79%) patients, and sodium-glucose co-transporter type 2 inhibitors (SGLT-2i) were received by 87 (42.4%) patients. The initial average dose of furosemide was 86 mg, and the average dose during hospitalization was 80 mg. A combination of diuretics of different classes was used in 32 (15.6%) patients.

Over a three-month period after discharge, 55 (26.8%) patients were rehospitalized for AHF. Mortality over three months was 5.4% (11 patients).

Table 1 presents the main characteristics of patients who required readmission within 3 months after discharge and patients who avoided hospitalization.

Among the patients who were readmitted within three months after discharge, most HAD de novo HF (39 (71%) vs. 72 (48%); *p* = 0.005), required increased dose of loop diuretics during hospital course (98 ± 46 vs. 82 ± 37; *p*—0.01) and at discharge (54 ± 41 vs. 41 ± 33; *p*—0.02). 9% of these patients received inotropic support during index hospitalization versus 1% in non-hospitalized group. Average EF % was consistent, as prevalence of HFrFE, HFmrEF, and HFpEF patients did not differ significantly between the groups as in cardiac and non-cardiac comorbidities.

The average follow-up period was 22 ± 12 months. All-cause mortality was 68 (33.2%), and cardiovascular mortality was 15.6% (32 patients).

**Figure 2:**
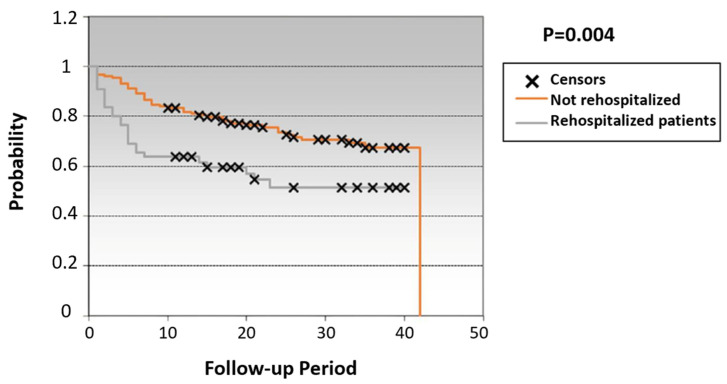
Kaplan–Meer curve showing the effect of rehospitalization within 3 months on the risk of death within 3 years.

The impact of early rehospitalization within 3 months on all-cause mortality is presented in Figure 2.

A stepwise multivariate regression analysis was performed including the following factors: age, gender, history of rehospitalization within the first 3 months after an episode of AHF, admission to the intensive care unit, history of MI, diabetes, AF, use of inotropic drugs during hospitalization, left ventricular end-diastolic dimension (LVEDD), LVEF, systolic blood pressure (SBP), diastolic blood pressure (DBP), heart rate and pulse pressure on admission, eGFR, NT-proBNP, blood sodium, and hemoglobin. As a result of this analysis, age (HR 1.04 [1.008–1.07]), heart rate on admission (HR 1.02 [1.004–1.03]), and the fact of rehospitalization within 3 months after the episode of AHF (HR 2.36 [1.2–4.7]) were found to be independent factors of an unfavorable prognosis.

## 4. Discussion

HF is the leading cause of hospitalization in general medical wards among patients over 65 years of age and is characterized by a high rate of rehospitalizations [4]. In the present study, we analyzed the prognostic impact of early rehospitalization that occurred during the 3-month vulnerable period after index hospitalization on 3 years follow-up.

Rehospitalization during first months after discharge is a common feature in HF due to complex nature of disease. Both disease-specific and patient-specific factors, mainly treatment adherence and healthcare system structure, have an effect on rehospitalization rate. Low socioeconomic status has been reported to be a predictor of increased HF readmissions. Patients living in areas with low neighborhood median household income (nINC) more often have more comorbid illness. In addition, patients in such neighborhoods are more often admitted to rural hospitals, which are less likely to offer comprehensive management programs. One study found that low-nINC patients with high comorbidity are rehospitalized at a higher rate than high-nINC patients with the same level of comorbidity (relative risk, 1.67; 95% CI, 1.01–2.76). However, treatment adherence, as it pertains to patients’ social or educational status, was not analyzed specifically in our study [7,8].

The fact of rehospitalization during the post-discharge “vulnerable” period has been a subject of ongoing investigation in heart failure research. Inclusion of 30-day all-cause readmission or death as a major focus of quality improvement and payment reform attests to the seriousness of readmission in patients with HF as a health economic problem. Many of these readmissions are predictable, and therefore, possibly preventable. As such, researchers and policy makers have placed significant focus on efforts to decrease readmissions and avoid excessive healthcare spending. In 2010, the USA introduced the Hospital Readmission Reduction Program (HRRP) under which hospitals with higher than expected readmission rates were penalized up to 3% of their annual Medicare reimbursements. On these grounds, it is important for clinicians to identify which patients may be at highest risk of being readmitted [9,10]. However, a universally accepted and clearly defined timeframe for this period remains elusive.

In a study by Vader et al. examining patient trajectories following an episode of AHF, the composite endpoint of rehospitalization or death occurred in 26% and 38% of patients within 30 and 60 days, respectively. Notably, the leading causes of hospitalization within the first 30 days were most frequently unrelated to heart failure decompensation [11].

Similar data were obtained as a result of the large American national study EVEREST, where the frequency of rehospitalizations in the 30-day period from the date of discharge was 24% [12].

In a study by Kitakata H, Kohno T, Kohsaka S et al., they evaluated the impact of early (0–30 days) and mid-term (31–90 days) readmissions on long-term outcomes. Their analysis demonstrated corresponding rehospitalization rates of 4.8% and 11.1% at 30 and 90 days, respectively [13].

In our study, 26.8% (55) of patients required rehospitalization for AHF within three months of discharge. This is comparable with the data reported by European countries and the United States but significantly higher than that in Japan.

The main determinants of readmission in previous publications were systolic blood pressure at admission, hyponatremia, chronic kidney disease stage, and heart rate [14,15].

In present study the patients who require readmission during 3 months after index discharge demonstrated necessity of increased doses of loop diuretics during hospital course and increased rate of inotropic support. Thus, it seems that the severity of congestion could be the determinator of rehospitalization rate [16]. Otherwise, this fact could highlight decreased response to diuretic therapy, a common problem observed in patients with AHF, which significantly limits therapeutic options [17]. In addition, high doses may be due to a history of HF and previous use of loop diuretics [18]. Less patients with de novo HF required early rehospitalization, which supports previous published data that hospitalization for AHF by itself had a high prognostic value for subsequent hospitalizations [19,20].

Notably, neither value of EF, nor specification for HFrEF, HFmrEF, or HFpEF status differed significantly between the groups. At the same time, increased left atrial dimension was demonstrated in the rehospitalized group. Dilation of cardiac chambers, including the LA, is a marker of remodeling and a known risk factor for cardiovascular events in patients with CHF [18]. Increased heart rate at index admission was found in the rehospitalized group, which supports previously published data [21,22].

We found that patients who required rehospitalization had lower hematocrit at discharge during index hospitalization. This can be due to a lower decongestion rate in these patients; however, current data is insufficient to support it.

In our study, we also analyzed the therapy when patients were discharged after an episode of decompensation of heart failure, and we found no differences between the groups that were rehospitalized during the first three months and those who were not. However, it is worth noting that the study was conducted at the very beginning of the introduction of angiotensin–neprilysin receptor antagonists into clinical practice in Russian city hospitals, and therefore we were unable to isolate such patients into a separate group. For the same reason, iSGLT-2 was used in only 42% of patients, and guanylate cyclase stimulants and myosin activators have not yet entered clinical practice in the Russian Federation. It is well known that these groups of drugs have helped significantly improve the prognosis of patients with heart failure (and here I will insert the link that this reviewer suggested to me) by influencing the neurohumoral system, but in our study it is not possible to assess the individual contribution of each of these drugs [23,24].

The main question of the study is if early rehospitalization had negative prognostic importance during long-term follow-up. We found that rehospitalization during the first 3 months increased mortality in univariate COX with RR of 1.86 [1.08–3.2] and multiple COX with RR of 2.36 [1.2–4.7], thus supporting the independent nature as an unbeneficial prognostic factor. Association between rehospitalization was analyzed in a few studies. Arundel et al. showed a link between early hospitalizations and increased mortality in these patients, but the recruitment of patients was conducted in 1998–2002, before the introduction of contemporary treatment [25]. In the work of Kitakata et al. (2020), it was shown that episodes of AHF with hospital admission both in the first 30 days and in the period 30–90 days after discharge are equally significantly associated with an increase in mortality from all causes during the subsequent 2 years of observation [13].

We believe that early rehospitalization could be a complex indicator for HF patients which combines disease-specific and patient-specific factors. Given the multifactorial and complex nature of hospitalization during vulnerable periods, we need to utilize modern healthcare technologies. Machine learning applications in healthcare are rapidly expanding. Machine learning-based prediction models can incorporate a large number of variables and could lead to better classification of patients with HF at risk for readmissions. As predictive models and quality of care measures improve, hospitals will be able to integrate these resources into better practice models to decrease readmission rates in patients with HF.

## 5. Limitations

This is an observational single-center study with all the typical limitations. Data were derived from municipal electronical medical records, and thus the data from those patients who attended private or other systems after discharge was incomplete. Treatment adherence as it pertains to patients’ social or educational status was not analyzed specifically. It is also worth noting the difference in the provision of medical care in Moscow and other regions of Russia. It is known that the average age of cardiology patients in Moscow is higher than in other regions of Russia. This is due to improved and more accessible medical care. Therefore, this data should be applied with caution to other regions and countries. Finally, the overall sample size of patients is relatively small, requiring larger studies to verify the results.

## 6. Conclusions

Patients who require rehospitalization within three months after discharge AHF are characterized by a history of previous hospitalizations for HF, the use of intravenous inotropic drugs, and higher doses of loop diuretics. Readmission for AHF within the first three months after discharge is an independent risk factor for mortality in patients with HF.

## Figures and Tables

**Table 1:** Comparative characteristics of patients who required readmission within 3 months after discharge and patients who avoided early readmission.

	Rehospitalized Patients	Not Rehospitalized	*p*-Value
Men, *n* (%)	26 (47)	90 (60)	0.09
Age, years	70 ± 13	72 ± 13	0.3
Previous MI (%)	32 (58)	76 (51)	0.36
De novo HF, *n* (%)	39 (71)	72 (48)	0.005
DM, *n* (%)	11 (20)	48 (32)	0.09
History of PCI/CABG/MABG, *n* (%)	11 (20)	29 (19)	0.93
History of AF, *n* (%)	42 (76)	106 (71)	0.46
AF on admission, *n* (%)	41 (74.5)	94 (63)	0.125
ICD/CRT/PM, *n* (%)	4 (7)	10 (7)	0.889
COPD, *n* (%)	10 (18)	34 (23)	0.475
Admission in intensive care unit, *n* (%)	22 (40)	53 (36)	0.56
Need for inotropic support, *n* (%)	5 (9)	2 (1)	0.007
LVEF, %	43 ± 16	43 ± 15	0.76
LVEF ≥50%, *n* (%)	21 (38)	62 (42)	0.659
LVEF 40–49%, *n* (%)	10 (18)	19 (13)	0.325
LVEF <40%, *n* (%)	24 (44)	68 (46)	0.8
LA diameter, cm	4.9 ± 0.8	4.6 ± 0.7	0.01
eGFR, mL/min/1.73 m^2^	55 ± 21	54 ± 22	0.62
Hemoglobin on admission, g/dL	125 ± 21	129 ± 25	0.29
Hematocrit at discharge	38 ± 6	41 ± 7	0.01
Blood albumin, g/L	37 ± 5	38 ± 5	0.05
Blood urea, mmol/L	9 ± 5	9 ± 4	0.79
Blood uric acid, µmol/L	490 ± 178	468 ± 164	0.42
Serum sodium, mmol/L	139 ± 4	140 ± 4	0.89
Furosemide dose during the first day of hospitalization, mg	98 ± 46	82 ± 37	0.01
Average dose of furosemide during hospitalization, mg	90 ± 41	77 ± 41	0.06
Combination diuretic therapy, *n* (%)	13 (24)	19 (13)	0.058
Furosemide dose at discharge, mg	54 ± 41	41 ± 33	0.02

AF = atrial fibrillation; CABG = coronary artery bypass grafting; CRT = cardiac resynchronization therapy; DM = diabetes mellitus; eGFR = estimated glomerular filtration rate; g/L = grams per liter; g/dL = grams per deciliter; ICD = implantable cardioverter defibrillator; MABG = coronary artery bypass grafting; mg = milligram; MI = myocardial infarction; LA = left atrium; PCI = percutaneous coronary intervention; PM = pacemaker.

## Data Availability

The authors confirm that the data supporting the findings of this study are available from the corresponding author upon reasonable request.
